# Prise en charge du rétrécissement urétral acquis: expérience du Service de Chirurgie Générale de Sikasso

**DOI:** 10.11604/pamj.2019.33.328.16724

**Published:** 2019-08-28

**Authors:** Salifou Issiaka Traore, Ousmane Dembélé, Amadou Maiga, Soumaila Traore, Aly Boubacar Diallo, Toure layes, Issa Diarra, Moussa Kante, Emmanuel Ballo

**Affiliations:** 1 Service Urologie, Service de Chirurgie Générale, Hôpital de Sikasso, Sikasso, Mali

**Keywords:** Urètre, rétrécissement urétral, dysurie, résection anastomose termino-terminale, Urethra, urethral stricture, dysuria, resection with termino-terminal anastomosis

## Abstract

Le rétrécissement urétral est une pathologie dont l'étiologie et la prise en charge varient selon le contexte. L'objectif de l'étude était de procéder à une analyse des aspects épidémiologiques, étiologique et thérapeutique du rétrécissement urétral dans notre contexte. Il s'agit d'une étude transversale longitudinale portant sur les cas de rétrécissement urétral acquis admis dans notre service entre mars 2014-février 2016. Age moyen: 24,5ans (10 et 81 ans). Le diagnostic fut confirmé par l'Uretro-Cystographie Rétrograde et Mictionnelle (UCRM). La longueur moyenne du rétrécissement était de 2,28cm (0,5-5cm). Les modalités thérapeutiques adoptées: la Résection Anastomose Termino-Terminale (RAT-T); la Dilatation Urétrale Rétrograde Itérative (DURI) etc. L'évaluation du résultat 6-15 mois postopératoire; satisfaisant en l'absence de récidive, RPM ≤30cc et un jet urinaire fort et mauvais en cas de récidive de dysurie ou RPM ≥100cc. Le RU occupe 7,14% de nos activités urologiques. La plus part de nos patients sont des cultivateurs provenant de la zone rurale. Les antécédents d'urétrite répétée ont été le plus souvent évoqués par nos patients et 78,57% d'entre eux étaient des hommes mariés dont 91% polygames. Les patients ont consulté surtout pour dysurie soit 50% de l'effectif et 50,01% de nos patients ce sont présentés avec une infection urinaire secondaire dont le germe prédominant est *Escherichia coli.* L'étiologie infectieuse a été la plus dominante soit 56,52%. La portion la plus atteinte a été l'urètre bulbaire soit 45,60% des cas. La RAT-T a été la technique la plus utilisée soit 39,13%. Globalement nous avons eu 85,65% de bon résultat et 13,04% d'échec; les taux de succès les plus élevés ont été obtenus avec la résection anastomotique soit 94,44% respectivement. Le rétrécissement urétral reste une pathologie fréquente chez les jeunes. L'étiologie infectieuse demeure la plus dominante dans notre contexte. L'accent doit être mis sur la prévention et la Prise en Charge (PEC) efficace et efficiente des Infections Sexuellement Transmissible (IST).

## Abstract (en)

Urethral stricture is a disease whose cause and management vary according to the context. This study aims to analyze the epidemiological etiological and therapeutic features of urethral stricture in our department. We conducted a longitudinal cross-sectional study of patients with acquired urethral stricture admitted to our department between March 2014 and February 2016. The average age of our patients was 24.5 years (10 and 81years). The diagnosis was confirmed by retrograde and voiding Urethro-Cystography (UCG). The average stricture length was 2.28cm (0.5-5cm). The therapeutic approaches included: resection with termino-terminal anastomosis; retrograde dilatation etc. Outcome assessment performed 6-15 months after surgery was satisfactory with absence of recidivism, PMR ≤30cc and strong urine flow and weak in the case of recurrence of dysuria or PMR ≥100cc. Urethral stricture accounted for 7.14% of our urologic treatments. Most of our patients were farmers from the rural area. A history of recurrent urethritis was most often reported by our patients and 78,57% of them were married men, among whom 91% were polygamous). The main reason for consultation was dysuria (50% of the study population) and 50.01% of our patients had secondary urinary tract infection, most commonly caused by Escherichia coli. The main cause of urethral stricture was an infection (56.52%). The most affected area was the bulbar urethra (45.60% of cases). UCG was the most used technique (39.13%). Overall outcomes were good (85,65%) and failure rate reached 13.04%; the highest success rate was achieved with resection with anastomosis (94.44% respectively). Urethral stricture is common among young people. Infection is the main cause in our department. Prevention is essential as well as an efficient and effective management of sexually transmitted infections.

## Introduction

Le Rétrécissement Urétral Acquis (RUA) se définit comme une diminution voire une oblitération du calibre, unique ou multiple et plus ou moins étendue du canal urétral, causé par une réaction fibro-sclereuse faisant suite à une agression d'origine infectieuse ou traumatique et qui gêne le libre écoulement des urines. Jadis, considéré comme principale complication de la blennorragie et des autres urétrites bactérienne atypique; nous avons espéré une diminution de sa prévalence avec l'avènement de l'antibiothérapie [1, 2]. Aujourd'hui, cette pathologie a tendance à devenir un problème d'actualité à cause du nombre de plus en plus croissant des accidents de la voie public et des manœuvres endo-urologiques intempestives [3]. Avec son taux d'échec et de récidive élevé, les controverses autour du choix de la technique opératoire la mieux appropriée pour obtenir un bon résultat à long terme, le rétrécissement urétral demeure un défi majeur Pathologie grave à cause du fait qu'elle est l'une des principales causes d'invalidité professionnelle temporaire, voire d'infirmité ou de mortalité parmi les adolescents et jeunes adultes qui constituent la tranche la plus active de la population africaine. Déterminer les aspects épidémiologiques et etiopathogeniques du rétrécissement urétral acquis à l'hôpital de Sikasso. Répertorier les motifs et circonstances de découverte du RUA de l'homme. Décrire les aspects cliniques, para cliniques et radiologiques du RUA. Décrire les différentes modalités de PEC du RUA chez l'homme et évaluer leur résultat.

## Méthodes

Il s'agit d'une étude transversale longitudinale prospective portant sur les cas de RUA de l'homme admis dans notre service de chirurgie générale de mars 2014 jusqu'en février 2016. Sont inclus dans cette étude tous les patients de sexe masculin, admis et Prise en Charge (PEC) pour rétrécissement urétral acquis dont le diagnostic a été confirmé par l'Uretro-Cystographie Rétrograde et Mictionnelle (UCRM) et acceptant de faire partir de l'étude. Exclus tous patients souffrant de rétrécissement urétral congénital, de tumeur maligne et les cas de vessie neurologique. Les patients sont opérés après stérilisation de l'urine sous anesthésie locorégionale en position Trendelenburg ou en position décubitus dorsal. Différentes modalités de prise en charge ont été adoptées. Evaluation du résultat 6-15 mois postopératoire succès: l'absence de récidive, résidu post mictionnelle inférieure à 50cc et maintien d'un jet urinaire fort après 1-2 séances de dilatation. Echec: dysurie, ré-sténose confirmée par UCRM et résidu post-mictionnel ≥100cc; faiblesse du jet après 3 séances de dilatation.

## Résultats

**Figure 1:**
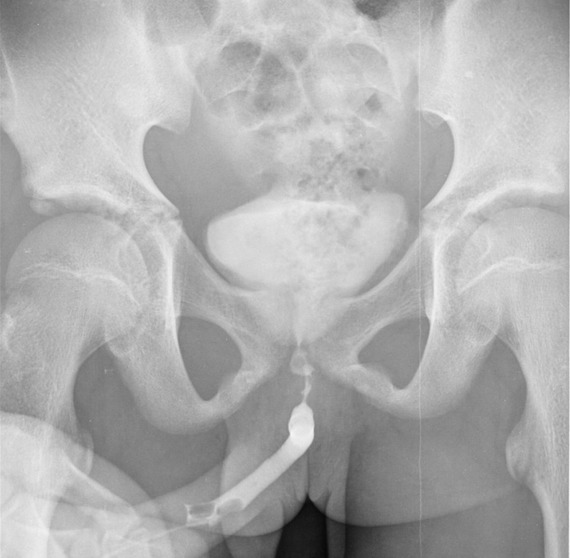
UCRM montrant un rétrécissement infectieux de l´urètre bulbaire

**Figure 2:**
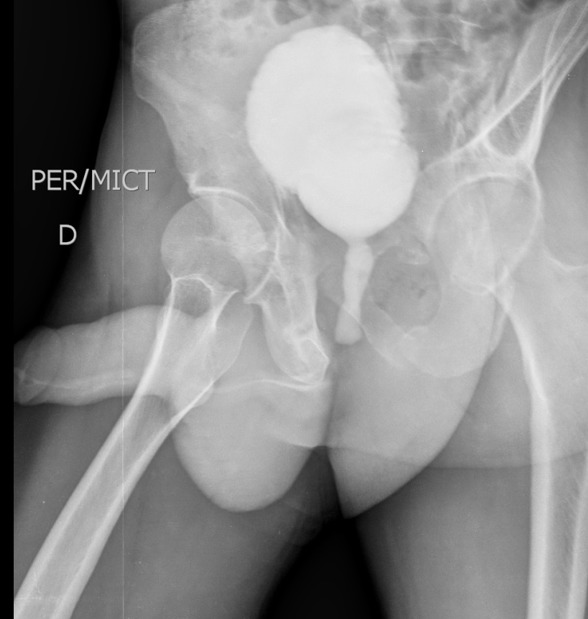
UCRM montrant un rétrécissement traumatique de l´urètre membraneux

Nous avons colligé au total 46 patients souffrant de RUA soit 7,14% de nos activités urologiques. Age moyen: 24,5 ans (10-81 ans). La tranche d'âge (21-30 ans) a été la plus représentée soit 30,95% des cas. Nos patients étaient surtout de nationalité Malienne (76,19%), suivie des Ivoiriens (23,80%); les ethnies les plus représentées étaient les Senoufos (46,7%) suivis des Peuhls (19,04%). Nos patients étaient surtout des illettrés (84,78%), exerçant le métier d'agriculteur à 47,61% et provenant majoritairement (64,28%) des communes rurales de Sikasso. 78,57% des patients étaient des hommes mariés dont 91% de polygame. Les patients ont sollicité une PEC pour diverse motifs dont la dysurie soit 50% suivie de la rétention aiguë d'urine et de l'incontinence d'urine (Tableau 1). Le délai moyen de Prise en Charge (PEC) est estimé à 458,4 jours (0,5-30 mois). La moitié de nos patients soit 50% ce sont présentés avec une complication infectieuse urinaire dont le germe le plus fréquent est *E. Coli* et qui est associée soit à une hyper créatininémie (26,06%) ou à un phlegmon péri-urétral/fistules urétro-cutanée (13,04%). 21,42% de nos patients ont évoqué une urétrite répétée comme principal antécédent. La part qu'occupent les rétrécissements traumatiques (Figure 1) secondaire à une lésion urétrale associée à une fracture du bassin est certes importante, mais le rétrécissement d'origine infectieuse illustré par l'atteinte du corps spongieux peri-uretral (Figure 2) reste la forme la plus dominante soit 56,52% (Figure 2).

**Tableau 1:** Répartition des patients selon le motif de consultation

Motif de consultation	Fréquence	Pourcentage
Rétention aigue d’urine	18	39,13%
Dysurie	23	50.0%
Incontinence urinaire	05	10,89%
**Total**	46	100%

**Figure 3:**
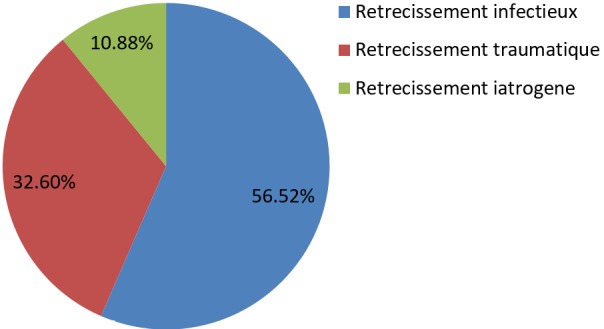
Répartition des patients souffrants de rétrécissement selon l´étiologie

La longueur moyenne du rétrécissement était de 2,28cm (0.5-5cm) et la portion urétrale la plus touchée a été l'urètre bulbaire soit 45,23% des cas (Tableau 2). RAT-T a été la technique la plus utilisée et pour maintenir un jet urinaire fort, elle a été après associée à une dilatation rétrograde dans 16,66% (Tableau 3). Globalement nous avons eu 85,95% de bon résultat et 13,04% d'échec (Figure 2, Figure 3). Les taux de succès les plus élevé ont été enregistrés avec la résection anastomotique termino-terminale et la dilatation urétrale rétrograde itérative soit respectivement 94,44% et 90% (P<0.05). Cependant, le plus fort taux d'échec a été enregistré avec les rétrécissements d'origine infectieuse soit 23,07%.

**Tableau 2:** Répartition selon le siège UCRM du rétrécissement

Siege du rétrécissement urétral		Fréquence	Pourcentage
Postérieur	urètre membraneux	11	23,80%
Antérieur	Bulbaire	21	45,65%
	Pénien	07	15,21%
	Méat urétral	02	4,76%
Mixte	Membraneux+bulbaire	05	11,90%
**Total**		46	100%

**Tableau 3:** Répartition des patients selon la modalité de prise en charge

Traitement chirurgical	Fréquence	Pourcentage
Dilatation antero-rétrograde	13	30,95%
Uretroplastie en 2 Temps (Ben Johnson)	1	2,38
Uretroplastie lambeau pédiculé (Orandi et onlay)	2	4,34%
Résection anastomose T-T	18	39,13%
Dilatation R adjuvant	03	16,66%
Méatotomie/Dilatation	2	4,76%
Dilatation rétrograde Itérative	10	21,73%
**Total**	**46**	**100**

## Discussion

La Prise en Charge (PEC) chirurgicale du rétrécissement urétral occupe 7,14% de nos activités urologiques; alors que K DJE et collègues [4] ont observé une prévalence hospitalière légèrement élevée. Cela pourrait s'expliquer par le fait que la disponibilité de prestation urologique spécialisée soit relativement récente et aussi par la timidité de nos activités endourologique. Age moyen: 24,5 ans avec prédominante (21-30ans). Chose qui est similaire au résultat d'Ouattara Z [5]. Ces résultats montrent que RUA est une maladie de l'adulte jeune, période où les gens sont beaucoup plus actifs aussi bien sur le plan sexuel que socioéconomique. La fréquence élevée d'hommes mariés avec une proportion assez importante de polygames, confrontée dans notre contexte à la prédominance des rétrécissements infectieux ainsi qu'à l'analphabétisme, met en exergue les difficultés pour une PEC efficiente des Infections Sexuellement Transmissible (IST).

Comme dans d'autres séries Africaines [6, 7], nous avons une proportion importante (56,52%) de rétrécissement d'origine infectieuse, confirmée par une prédominance de l'atteinte bulbaire, zone où le cul de sac bulbaire et la densité élevée des glandes péri-urétrales favorisent la stagnation et la prolifération des germes. Nous constatons une certaine conformité entre nos résultats et la littérature, où la forte prévalence de la forme compliquée des rétrécissements sclerospongieux en milieu tropical africain résulte très souvent d'un retard de PEC, retard du probablement aux difficultés d'accès en soins spécialisés, à l'automédication et aux croyances traditionnelles néfaste. Alors que, cause du niveau de développement socio sanitaire assez élevé et de leur facilité d'accès aux soins, ce type de rétrécissement est présentement rare en milieu occidental, contrairement à ceux d'origines traumatiques et iatrogènes [8, 9].

Selon la littérature [10] 18-72% des rétrécissements urétraux secondaires à une fracture du bassin sont associés à des troubles sexuels. Paradoxalement, nous n'avons enregistré aucun cas de postopératoire. Dans notre série, de telles plaintes proviendraient surtout des rétrécissements iatrogènes associés souvent à d'autres comorbidité et dont la PEC n'a pas toujours été agressive. Chose qui serait due au fait que, nous avons systématiquement effectue une dérivation urinaire temporaire chez tous nos patients dont le traumatisme du bassin se trouve associés à des signes d'atteinte urétrale, donnant ainsi le temps aux tissus de se reconstituer avant l'intervention. La DURI est une des plus vieilles techniques, utilisée comme traitement primaire ou adjuvant du rétrécissement urétral et qui reste efficace, à condition que l'indication soit bien posée et que ces principes soient respectés. En effet, la dilatation rétrograde itérative donne de très bon résultat lorsqu'il s'agit de rétrécissements perméables, courts et uniques dont l'atteinte concerne très souvent la muqueuse, le tissu spongieux généralement épargné. Avec (94,44%) de bon résultat, conformément à la littérature [11, 12] la RAT-T apparait comme la meilleure technique, quelque soit l'étiologie. Mais le délai minimum de 3 mois post traumatique doit être respecté dans les rétrécissements traumatiques.

## Conclusion

Le Rétrécissement Urétral Acquis (RUA) est une Pathologie fréquente, surtout chez les jeunes et les adultes. L'étiologie infectieuse demeure dominante dans notre contexte; seul la prévention et la prise en charge efficace des IST sans oublier la/ou les partenaires s'avèrent nécessaires. Le choix de la modalité thérapeutique doit toujours commencer par les plus simples et moins invasives (DURI) afin de mettre à profit toutes les potentialités. La Résection Anastomose Termino-Terminale (RAT-T) par abord périnéal ou mixte est la technique la plus efficace pour la Prise en Charge (PEC) des rétrécissements urétraux, quelque soit l'étiologie; mais un délai de 3 mois post-traumatique doit être respecté concernant les rétrécissements associés à une fracture du bassin.

### État des connaissances actuelles sur le sujet

- Le rétrécissement d'origine infectieuse était Jadis prédominant dans notre contexte, à cause du manque d'éducation en matière de santé de la reproduction, des difficultés d'accès aux soins spécialisés et à l'insuffisance de l'arsenal antibiotique;
- Aujourd'hui, à cause du nombre de plus en plus croissant des accidents de la voie public et des manœuvres endo-urologiques, cette pathologie a tendance à devenir un véritable problème de santé public;
- Controverses et défi autour du choix de la modalité thérapeutique la mieux appropriée pour obtenir un bon résultat à long terme.

### Contribution de notre étude à la connaissance

- Malgré l'augmentation du niveau de développement socio sanitaire et la forte disponibilité d'agent antiinfectieux, la prévalence du rétrécissement infectieux demeure toujours élevée;
- Cette forte proportion des rétrécissements d'origine infectieuse pourrait s'expliquer par les difficultés de Prise en Charge (PEC) efficiente des IST dans un contexte où l'analphabétisme, l'automédication et la polygamie sont toujours d'actualité;
- La Résection Anastomose Termino-Terminale (RAT-T) par abord périnéal ou mixte est la technique la plus efficace pour la PEC des rétrécissements urétraux; à condition qu'un délai de 3 mois post-traumatique soit respecté concernant les rétrécissements associés à une fracture du bassin.

## Conflits des intérêts

Les auteurs ne déclarent aucun conflit d'intérêts.
